# Adapting a Phage to Combat Phage Resistance

**DOI:** 10.3390/antibiotics9060291

**Published:** 2020-05-29

**Authors:** Elina Laanto, Kati Mäkelä, Ville Hoikkala, Janne J. Ravantti, Lotta-Riina Sundberg

**Affiliations:** 1 Faculty of Biological and Environmental Sciences, Molecular and Integrative Biosciences Research Programme, University of Helsinki, 00014 Helsinki, Finland; janne.ravantti@helsinki.fi; 2 Department of Biological and Environmental Science, Nanoscience Center, University of Jyvaskyla, 40014 Jyvaskyla, Finland; kati.j.makela@jyu.fi (K.M.); ville.hoikkala@jyu.fi (V.H.); lotta-riina.sundberg@jyu.fi (L.R.S.)

**Keywords:** coevolution, fish pathogen, phage resistance, phage therapy

## Abstract

Phage therapy is becoming a widely recognized alternative for fighting pathogenic bacteria due to increasing antibiotic resistance problems. However, one of the common concerns related to the use of phages is the evolution of bacterial resistance against the phages, putatively disabling the treatment. Experimental adaptation of the phage (phage training) to infect a resistant host has been used to combat this problem. Yet, there is very little information on the trade-offs of phage infectivity and host range. Here we co-cultured a myophage FCV-1 with its host, the fish pathogen *Flavobacterium columnare*, in lake water and monitored the interaction for a one-month period. Phage resistance was detected within one day of co-culture in the majority of the bacterial isolates (16 out of the 18 co-evolved clones). The primary phage resistance mechanism suggests defense via surface modifications, as the phage numbers rose in the first two days of the experiment and remained stable thereafter. However, one bacterial isolate had acquired a spacer in its CRISPR (Clustered Regularly Interspaced Short Palindromic Repeat)-Cas locus, indicating that also CRISPR-Cas defense was employed in the phage-host interactions. After a week of co-culture, a phage isolate was obtained that was able to infect 18 out of the 32 otherwise resistant clones isolated during the experiment. Phage genome sequencing revealed several mutations in two open reading frames (ORFs) likely to be involved in the regained infectivity of the evolved phage. Their location in the genome suggests that they encode tail genes. Characterization of this evolved phage, however, showed a direct cost for the ability to infect several otherwise resistant clones—adsorption was significantly lower than in the ancestral phage. This work describes a method for adapting the phage to overcome phage resistance in a fish pathogenic system.

## 1. Introduction

Phage therapy is a widely recognized and studied method for combating bacterial pathogens. It is gaining increased attention as an alternative or addition to antibiotic treatment, due to the global crisis of emerging antibiotic resistance. However, subjecting bacteria to phages raises concerns of phage resistance in pathogenic bacterial populations. Bacteria can use either passive (mutations in the phage receptors) or active (e.g., restriction-modification and Clustered regurarly Interspaced Short Palindromic Repeats (CRISPR)/CRISPR-associated protein (Cas) defense, actively degrading phages) mechanisms to prevent the phage infection, see, e.g., [1,2]. However, phage resistance is often costly for the bacteria and may cause loss of virulence, e.g., [3,4,5], suggesting that locally appearing phage resistance might not be a problem. On the other hand, the continuous arms race between bacteria and phage also assures the rise of phage progeny that have evolved to infect the resistant bacteria. Phage can quickly adapt by changing a part or parts of their route of infection and/or bypass active resistance mechanism via genomic mutations or directly inhibiting CRISPR-Cas defense [6,7]. Using phage-bacterium coevolution as a tool could be an important part in developing phage therapy.

Methods for phage training have been developed for selecting evolved phages that are better suited for phage therapy purposes. The earliest phage training method, called Appelmans experiment, originates from the early 1920s and has been reviewed recently [8]. Other examples include co-culture of bacteria and phage and serial passage of phage. Such experiments with *Pseudomonas aeruginosa* and its phages [9,10] resulted in evolved phages that were more efficient in reducing bacterial densities. *Flavobacterium columnare,* the causative agent of columnaris disease in fish, is a severe pathogen in freshwater fish farming worldwide causing major economic losses [11]. We have previously tested phage in the treatment of, as well as as a prophylactic against, columnaris disease with promising results. In *F. columnare* phage resistance evolves mainly through surface modifications, which results in fitness costs via loss of motility and virulence [12,13]. The cells of *F. columnare* form different colony morphologies under laboratory conditions that differ in their ability to glide on surfaces and cause disease. Phage resistance is seen as a change in colony morphology from rhizoid (Z, expression of gliding motility) to rough (R, non-gliding) [12,13] and similar connections have been seen in other model systems [14]. In naturally evolving *F. columnare* populations, surface resistance has not been detected, although data from the coevolving phage population indicate counter-adaptations to such resistance mechanisms [15].

However, it is still unclear how the phage treatment would affect phage susceptibility in the case of *F. columnare*—would there be a problem with phage resistance? Previously, a controlled experiment on the coevolution of *F. columnare* and phage was performed to study the hosts CRISPR-Cas spacer acquisition [16]. Here, we aimed to understand phage response during coevolution and the cost of ability to infect wider range of the otherwise resistant bacterial clones in phage phenotype. The occurrence of phage resistance and phage ability to overcome the developed host resistance was studied with phage-host pair originating from our previous study, host strain B245 and a myophage FCV-1 with a linear 47 kbp genome [15,17]. Host and phage were incubated for a four-week period in lake water without nutrient additions. It is known that cells of *F. columnare* survive in lake water for at least a 5-month period [18]. Phages infecting *F. columnare* isolated thus far do not have a broad genetic or morphological variety [15,17]. Hence, one option is that *F. columnare* could be combated more efficiently with evolved, broad-spectrum host range phages.

During the experiment, one evolved phage isolate was selected for detailed characterization because of its capability to infect most of the bacterial clones isolated that were resistant to the ancestral phage. The cost for the ability to overcome phage resistance was detected in weaker adsorption. We pinpoint the molecular level changes in the evolved phage genome. The method is a simple one and could be used to develop evolved phages within this system and our results encourage the use of experimental evolution in producing phages for phage therapy applications.

## 2. Results

### 2.1. Bacteria and Phage during the Experiment

**Figure 1:**
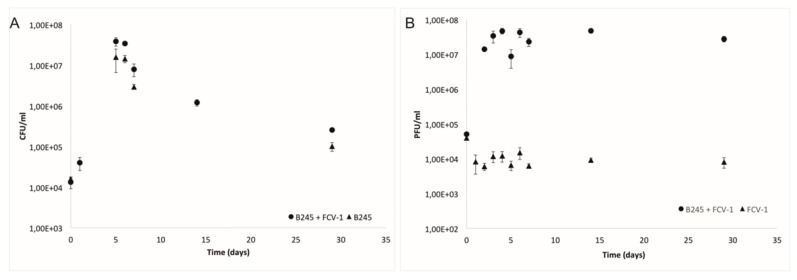
(**A**) Mean and standard deviation of colony forming units per mL (CFU/mL) of three replicates of *Flavobacterium columnare* strain B245 during the four-week experiment with phage FCV-1 and without phage. (**B**) Mean and standard deviation of plaque forming units per ml (PFU/mL) of phage FCV-1, with B245 and FCV-1 without bacteria.

Monitoring of the phage and bacterial populations during the 29-day experiment (Figure 1A) showed that in the first two days, bacterial cell numbers reached the highest concentration in both treatments, with (PB) and without the phage (B). Additionally, the phage titer (PFU/mL) increased approximately three logs (from 10^4^ PFU/mL to 10^7^ PFU/mL) during these first days, remaining stable until the end of the experiment. In the phage only treatment, the phage numbers remained stable throughout the experiment (Figure 1B).

### 2.2. Phage Resistance Detected by Morphotype and CRISPR Spacer Acquisition

**Figure 2:**
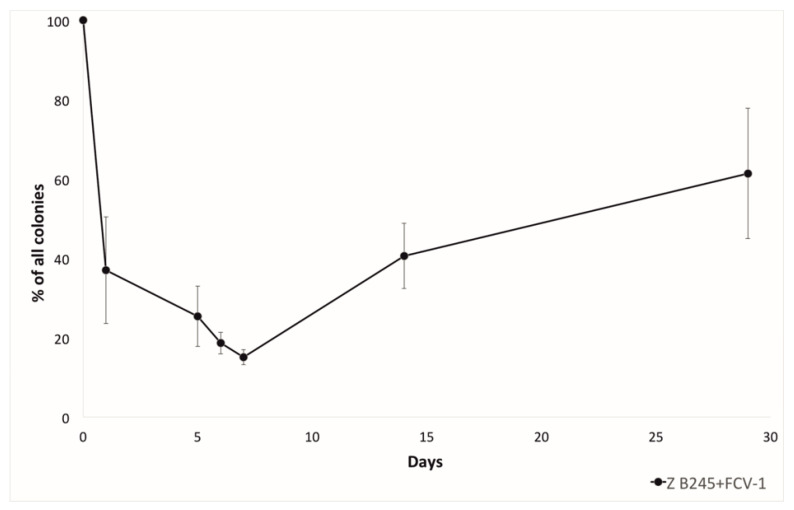
The proportion (percentage) of rhizoid (Z) colony types out of all of the colonies (rhizoid and rough) during the lake water experiment from the coculture of *F. columnare* strain B245 and phage FCV-1. In the treatments without phage, rough colonies were seen only on the last day (29) of sampling.

*F. columnare* evolves resistance against phage mainly via surface modifications, which cause a colony morphology change from the ancestral rhizoid (Z) to rough (R) [12]. Thus, the proportion of rhizoid (generally sensitive for phage) and rough (generally resistant to phage) colonies during the experiment was also monitored. Here, the phage-exposed bacteria formed more rough colonies starting from the first day, and the proportion of rough colonies was highest after 7 days of co-culture. At the 14^th^ day, sampling the proportion of rhizoid colonies had again increased, and at four weeks, the proportion of rhizoid colonies was higher than the rough colonies (Figure 2). The two CRISPR arrays of *F. columnare*, both of which contain pre-existing phage-targeting spacers [15], were checked for acquisition of new spacers during the co-culture. From 65 bacterial isolates that were tested, one isolate from two weeks of co-culture had acquired an additional spacer in its RNA targeting type VI-B array (5′-CATTGCAATGAGTGGATAGATGTGCAATG-3′). The spacer crRNA matched the putative large subunit terminase on the non-coding strand of FCV-1 genome and was thus not complementary to the predicted mRNA of this gene. Inability to bind RNA is in contrast with the other pre-existing FCV-1 targeting spacers in this locus that are invariably complementary to mRNA.

### 2.3. Phage Infectivity against the Evolved Bacteria

**Figure 3:**
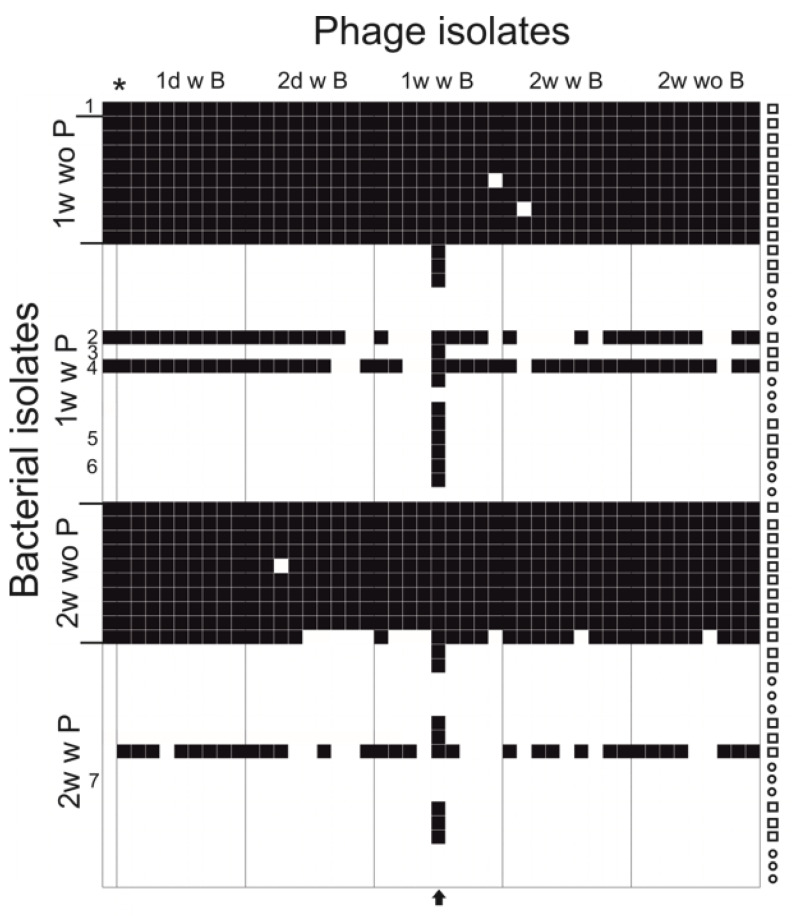
Infectivity of the ancestral phage and phage isolates from one week and two weeks against the original bacteria and isolates from one week and two weeks. Black squares indicate infection and white squares indicate no infection. Colony morphology of the bacterial isolate is indicated on the right, where a square indicates rhizoid and a circle indicates rough. * = Ancestral phage. Numbers correspond the strains in Table 1 (1 being the ancestral strain B245). An arrow at the bottom indicates the evolved phage FCV-1.01. B = bacteria, P = phage, w = with, wo = without.

Infectivity of co-evolved (and ancestral phage) phage isolates (45 isolated phage plaques) from the experiment was tested against all the bacterial isolates (including ancestral host) collected during the experiment (55 bacterial clones), including rhizoid and rough morphotype colonies from phage and bacteria (PB) and bacteria alone (B) treatments (Figure 3). In general, the clones from bacteria alone were all sensitive to all phage isolates, and clones from co-cultivation were resistant. The number of generally resistant clones was 32 out of the 35 tested. One of the triplicates demonstrated different interaction dynamics, since bacterial isolates were detected that were sensitive to most of the phage isolates.

One phage isolate differed from other phage isolates by being able to infect 41 out of the 55 tested bacterial isolates, whereas the other phage isolates from the experiment infected approximately 20 out of 55 isolates. In addition, the evolved phage was able to infect 18 out of the 32 otherwise-resistant bacterial clones. The titers of the ancestral FCV-1 and this evolved phage FCV-1.01 were compared on the original host, colony variants and selected isolates from the experiment (Table 1). The evolved phage FCV-1.01 was able to infect all while the original FCV-1 was able to infect one of the rhizoid isolates from week one (in addition to original host).

### 2.4. Ability to Infect Phage Resistant Clones Has a Direct Cost on Phage Adsorption

**Figure 4:**
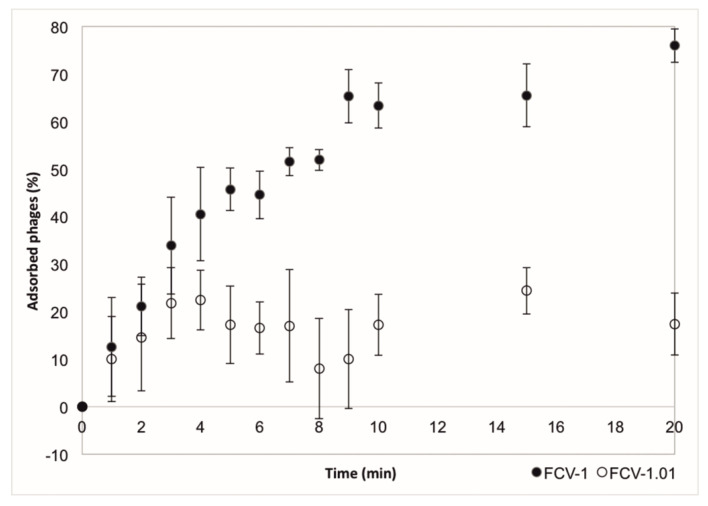
The mean and stdev of a typical adsorption as percentage (%) of adsorbed phage in time (minutes) of the original FCV-1 compared to the evolved FCV-1.01. Experiment was performed in triplicates.

The adsorption of the ancestral and evolved phage to the cells was measured using the host C4 soft colony type. Evolved phage had a weaker adsorption than the ancestral phage FCV-1 (Figure 4). In 10 min, approximately 63% of the ancestral phage had been adsorbed while for the evolved phage the amount was approximately 17%. We aimed to determine other life cycle parameters of both phages as well but were not able to replicate the experiments.

### 2.5. Nucleotide Level Differences Show Changes in Putative Tail Proteins

**Figure 5:**
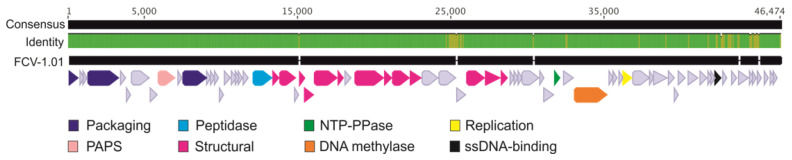
Genome alignment of the ancestral FCV-1 (accession number KY979235.1) and the adapted phage FCV-1.01 (accession number MT431535) displaying the genome consensus and FCV-1.01 with predicted open reading frames (ORFs) as arrows. Identity is displayed as green (100% identity) and yellow (the locations of differences between the two genomes). Breaks in the FCV-1.01 bases indicate the locations of deletions compared to FCV-1. Putative functions are shown in colors indicated in the bottom and the light blue arrows indicate the hypothetical proteins. PAPS stands for phosphoadenosine phosphosulfate sulfurtransferase, NTP-PPase for nucleoside-triphosphatase.

Sequencing of the evolved phage enabled a comparison to the ancestral phage genome in nucleotide level (Figure 5). Interestingly, some of the mutations in the evolved phage are in the same area as in phages that have been previously isolated from aquaculture setting [15]. Mutations were concentrated in the 25 kbp area of the 46 kbp long genome, and as was also seen in the phages from nature, the left end of the genome was more conserved, while the right end was more variable. The two ORFs (ORFs 35 and 36) in the 25 kbp are in between several putative tail genes and are most likely responsible for encoding parts of the tail structure. However, no function can be predicted due to unreliable hits in BLASTp and HHPred. In addition, most of the ORFs at the right end of the genome are missing a putative function but there are ORFs that are putatively responsible for DNA metabolism (e.g., ssDNA binding protein).

## 3. Discussion

Bacteria and phage are in a continuous arms race [2,19,20]. For phage therapy to become a reality, many aspects of the coevolution need to be resolved before adding phages to, e.g., aquaculture systems. The abundant use of chemical treatments over decades has caused the ongoing antibiotic resistance crisis, demonstrating that eradicating evolving biological material (bacteria) requires well-planned methods and thorough risk analysis. The applicability of phage in preventing and treating bacterial diseases has been shown to be efficient, and well-designed phage therapy provides a serious tool for combatting antibiotic-resistant bacteria [21]. Prevention of columnaris disease (causative agent *F. columnare*) with phage has been tested previously [22,23]. Here, we focused on the phage resistance in *F. columnare* and characterized the counter-adaptation by the phage. We detected one phage isolate that was able to overcome most of the evolved resistance. The cost for the ability to infect the otherwise-resistant bacterial clones was detected as weaker adsorption in the evolved phage.

Previously, we showed the molecular level detail on the phage-bacterium coevolution under semi-natural fish farming conditions over the course of several years [15]. The present study was a natural continuation for the field data, and we used the same host and phage isolate but followed the coevolution in a short time frame in a closed system. We were able to detect a rapid increase in both phage and bacterial population sizes that then remained stable for the rest of the experiment. There were only small fluctuations in phage and bacterial numbers after the first rapid rise, suggesting passive primary resistance in bacteria. Lake water was not filtered for the experiment and particles for biofilm growth were present that could promote phage production and susceptibility as was seen with addition of mucin [23]. Here, we did not add resources, which would have likely reduced the cost in terms of resistance mutations [24,25,26,27]. However, one bacterial clone was isolated after two weeks of co-culture with an added spacer in its type VI-B CRISPR-Cas locus spacer array. Indeed, spacer acquisition events in both identified CRISPR-Cas loci of *F. columnare* have been described [16]. Here, the addition of spacer indicates that a small proportion of the bacterial population was still sensitive for phage allowing active CRISPR-Cas spacer acquisition. Since the phage pressure outside the cells was high, it is supposed that the cells rely on other defense mechanisms rather than CRISPR-Cas, while CRISPR-Cas could be a way to drive the phage population to extinction [28]. In addition, the type VI-B CRISPR-Cas is likely to be involved in cellular dormancy, thus preventing phage production [29].

In *F. columnare* the change of colony morphology is spontaneous, but also inducible by starvation [18] and the presence of phage [12]. When incubated together with phage in lake water, the proportion of rough colonies started to rise among the rhizoid. After one week of incubation, the proportions stabilized to approximately 50% of each. This also suggests that the primary defense of this bacterium in these conditions is to alter the gliding motility machinery and surface structures. The information of the phage receptors in *F. columnare* is missing, but they are likely to be part of the gliding motility machinery, as has been proposed in *F. johnsoniae* [30,31]. It seems that the rough colony type is costly for the bacteria, as it is prone to revert back to the rhizoid type, supporting the fact that only the rhizoid type is isolated from the nature and fish farms [32].

While testing the infectivity of the phage isolates obtained during the co-culture, one isolate from 7th day time point was significantly more able to infect the otherwise resistant bacterial clones. The evolved phage was able to infect some of the rough isolates, despite the common phage resistance displayed by this colony type [12,33]. The efficiency of plating of the ancestral and the evolved phage was compared against some of the bacterial clones isolated from 7^th^ day and 14^th^ day time points. The evolved phage was equally efficient in producing plaques on all of the bacteria except for a slight drop in titer on one rough colony type isolate. The ancestral phage FCV-1 infected only the ancestral host and, with lower efficiency, one rhizoid isolate. The isolate with an extra spacer was resistant to the ancestral FCV-1.

Sequencing of the evolved phage genome indicated especially two ORFs (ORF35 and ORF36) involved in the ability to infect the otherwise resistant bacterial clones due to several nucleotide differences compared to the ancestral phage. No function for these ORFs was predicted, but their position in the tail module, between the tail proteins (putative baseplate and distal tail protein), suggests that they are tail proteins. The same ORFs were suggested to be related to host recognition according to the genomic data from a long-term study from nature [15]. The most understood phage tail structure is that of phage T4 [34,35]. In T4, the long tail fiber is a product of four different genes (and requires additional protein products to function). It interacts with both OmpC and LPS on the host surface, receptors that are used for host binding and moving on the surface of the cell to find a suitable site for infection. Previously, it has been shown that mutations especially in the gp37 of the tail fiber are responsible for adsorption specificity in T4 [36]. It is likely that ORF35 and ORF36 are part of tail fiber in FCV-1 and function as host recognition and/or attachment and mutations in these genes result in ability to infect wider range of the bacterial clones and affects the adsorption efficiency, as the genetic data can be associated with the phage phenotypic data. We observed a difference in the adsorption of the ancestral and evolved phage. A cost of the ability to infect wider range of the bacterial clones was detected as lower adsorption of the evolved phage. In contrast, Sergueev et al. [37] obtained an adapted phage in methicillin-resistant *Staphylococcus aureus* (MRSA) with similar adsorption to sensitive and resistant strains as the ancestral phage, indicating other phage resistance mechanisms than cell surface modifications. While it is known that small proportions of phage populations have an exceptionally low adsorption rate suggested to function as a survival strategy [38], the low adsorption in FCV-1.01 is likely to be the result of a cost from the evolved ability to infect wider range of the otherwise resistant bacterial clones.

It seems that the aquatic environment and the interaction of phage and fish mucus together with the external nature of the columnaris disease provide a promising ground for developing phage therapy in this system for aquaculture settings. However, the observed diversity within the thus far isolated phages infecting *F. columnare* has been limited [15,17], hampering the development of phage cocktails containing several types of lytic phages. Thus, phage adaptation and expansion of the ability to infect wider range of the bacterial clones could be future methods to combat the pathogen as has been described, for example, in phage T7 [39]. In addition, host range can be engineered synthetically by designing targeted variations in receptor binding proteins [40].

Although our approach to studying coevolution is an oversimplification compared to the environment at the fish farm and the ways the system would evolve in the fluctuating environment with the pathogen host (fish) present, the approach presented here could still have applicability in preparing phage cocktails, as has been done in phage therapy since the beginning of phage research [8]. Additionally, we see that the phage resistance countered by the phage with mutations especially in ORFs 35 and 36. However, the reduced adsorption efficiency might select against this phage in a real-life system and is possibly detected here only because of the static environment. These results suggest that it is important to monitor the trade-offs of increased ability to infect a wider range of the bacterial clones versus phage life cycle in the case of producing variants in laboratory conditions. Whether the phage isolated in this study could also be more efficient in phage therapy, as was shown for *Pseudomonas aeruginosa* [10], remains to be studied. In case the evolved phage would be more virulent it would be feasible to evolve such phages for phage cocktails.

## 4. Materials and Methods

### 4.1. Bacteria and Phage

Bacteria and phage used in the study have been described previously; see [17] for phage FCV-1 and *F. columnare* strain B245. Furthermore, we used strain C4, a soft colony morphotype variant [41], for phage life cycle experiments, as the phage propagation has not been successful in liquid with other strains, although phage forms plaques on the soft agar overlay. Shieh [42] medium was used in all cultivations with 1% agar (Thermo Fisher Scientific, Waltham MA, USA) in plates and 0.7% in overlay agar.

### 4.2. Coevolution Experiment in Lake Water

Lake water was collected from the surface layer of a boreal lake Leppavesi in Central Finland (N 62.267670, E 25.910923), autoclaved for 20 min at 121 °C, and stored at +4 °C before use. Phage and bacteria (PB), only bacteria (B) and only phage (P) were inoculated in 40 mL of lake water in triplicates. Bacterial density was adjusted to 1 × 10^4^ CFU/mL, and phage was added to multiplicity of infection (MOI) of 1. The cultures were maintained under constant agitation (50 rpm) throughout the experiment (29 days) at 23 °C. Both bacteria and phage were sampled from each treatment and CFU/mL and PFU/mL were determined by standard plating methods.

### 4.3. Sampling

Both bacterial (CFU/mL) and phage (PFU/mL) numbers were measured and the distribution of rhizoid (Z) and rough (R) colony morphotypes was monitored. Single colonies of both morphotypes (three of each in each replicate) were picked from PB and B and cultivated in 1 mL Shieh and stored in 10% glycerol (Sigma Aldrich, Saint Louis, MO, USA) and 10% FCS (Thermo Fisher Scientific, Waltham, MA, USA) in −80 °C for further analysis (total of 360 colonies throughout the experiment). Single plaques were picked into 500 μL Shieh from PB and B and stored in 10% glycerol in −80 °C.

### 4.4. Infectivity of Isolated Phage

Evolved phage plaque isolates (from four time points; one and two days and one and two weeks from PB and two weeks from B) were tested against bacterial isolates from PB and B from one week and two weeks, three isolates per replicate from B, and six isolates per replicate from PB, resulting in 45 phage isolates against 55 bacterial isolates (including ancestral phage and bacteria). One milliliter of o/n grown bacteria was added to 9 mL of soft agar and plated on top of an agar plate. Plaque lysates were spotted on the bacterial lawn as such and in ten- and hundred-fold dilutions for the detection of individual plaques. On the selected strains, phage infectivity was measured by mixing 100 μL of ten-fold dilutions of phage lysate with 300 μL of o/n grown bacteria in 3 mL of soft agar and poured on an agar plate.

### 4.5. Adsorption Test

Adsorption of the original FCV-1 and evolved phage isolate FCV-1.01 was compared using *F. columnare* strain C4 as a host. The adsorption tests were performed following the procedure of Kropinski [43] in triplicates. In brief, bacteria were grown to logarithmic phage, diluted and divided into 9 mL cultures. Medium without bacteria was used as a control. Approximately 3 × 10^5^ PFU was added in 1 mL volume. Samples (50 µL) were taken to eppendorf tubes with 950 µL of precooled medium including chloroform (VWR, Radnor, PA, USA). Number of free phages was determined from the plaque forming units and used for calculating the percentage of adsorbed phages.

### 4.6. Sequencing of the Phage Genome

Genome of the evolved phage FCV-1.01 was extracted and sequenced and compared to the original FCV-1 [15]. In brief, filtered phage lysate was treated with RNase and DNase and phages were precipitated using ZnCl_2_ (40 mM), pelleted and resuspended to TES-buffer (0.1 M Tris-HCl pH 8; 0.1 M EDTA; 0.3% SDS). Protease K was used for the release of genomic DNA which was purified using columns from DNeasy Blood&Tissue Kit (Qiagen, Hilden, Germany). Sequencing was done commercially with Illumina MiSeq at the sequencing unit of Institute for Molecular Medicine Finland (FIMM). The phage genome was assembled using Velvet-assembler (v. 1.2.10) with optimal k-mers per genome (average coverage ~ × 1600). Genomes of ancestral FCV-1 and evolved FCV-1.01 were aligned using MUSCLE [44] using default settings suggested by Geneious 7.1.4 (Biomatters Ltd., Auckland, New Zealand).

### 4.7. CRISPR Spacer Array Sequencing

In total, 65 bacterial isolates were checked for possible spacer additions in their type II-C CRISPR locus (CRISPR1) and in type VI-B locus (CRISPR2) using PCR and Sanger sequencing as described earlier [15]. In brief, CRISPR loci were amplified using the Phusion Flash II polymerase (Thermo Fisher Scientific, Waltham, MA, USA). The annealing temperature was 65 °C for CRISPR1 and 67 °C for CRISPR2, and the elongation step was 30 s for CRISPR1 and 27 s for CRISPR2. Purified PCR products were sequenced with BigDye^®^ Terminator v3.1. Cycle Sequencing kit using 3130xl Genetic Analyzer (both from Applied Biosystems, Foster City, CA, USA). Two replicates were done for each read and the consensus sequences were manually determined using Geneious 9.1.4 (Biomatters Ltd., New Zealand).

### 4.8. Data Availability

The sequenced phage FCV-1.01 genome was deposited in Genbank under the accession number MT431535.

## Figures and Tables

**Table 1:** Titers (PFU/mL) of ancestral FCV-1 and evolved FCV-1.01 on selected isolates (number in bold before strain corresponds the bacterial strains marked in Figure 3).

	Phage Titer (pfu/mL)	
Bacteria	FCV-1	FCV-1.01
C4	1,1 × 10^10^	1,1 × 10^10^
**1** ancestral B245	1,3 × 10^10^	1,0 × 10^10^
**2** (1 week), sensitive^1^ rhizoid	6,0 × 10^7^	1,4 × 10^10^
**3** (1 week) resistant^1^ rhizoid	*	1,1 × 10^10^
**4** (1 week), resistant^1^ rough	NA	1,0 × 10^10^
**5** (1 week), resistant^1^ rhizoid	**	1,1 × 10^10^
**6** (1 week), resistant^1^ rough	NA	4,9 × 10^09^
**7** (2 weeks), resistant^1^ rhizoid, spacer added	0	2,0 × 10^10^

^1^ resistant or sensitive to most of the phage isolates, * inhibition of growth (but no plaques) was detected in 1000-fold dilution, ** inhibition of growth (but no plaques) was detected in 10-fold dilution, NA = not assigned.

## References

[1] Rostøl J.T., Marraffini L. (2019). (ph)ighting phages: How bacteria resist their parasites. Cell Host Microbe..

[2] Hampton H.G., Watson B.N.J., Fineran P.C. (2020). The arms race between bacteria and their phage foes. Nature.

[3] Heierson A., Sidén I., Kivaisi A., Boman H.G. (1986). Bacteriophage-resistant mutants of Bacillus thuringiensis with decreased virulence in pupae of Hyalophora cecropia. J. Bacteriol..

[4] Capparelli R., Nocerino N., Iannaccone M., Ercolini D., Parlato M., Chiara M., Iannelli D. (2010). Bacteriophage therapy of Salmonella enterica: A fresh appraisal of bacteriophage therapy. J. Infect Dis..

[5] Friman V.-P., Hiltunen T., Jalasvuori M., Lindstedt C., Laanto E., Örmälä A.-M., Laakso J., Mappes J., Bamford J.K. (2011). High temperature and bacteriophages can indirectly select for bacterial pathogenicity in environmental reservoirs. PLoS ONE.

[6] Samson J.E., Magadán A.H., Sabri M., Moineau S. (2013). Revenge of the phages: Defeating bacterial defences. Nat. Rev. Microbiol..

[7] Hwang S., Maxwell K.L. (2019). Meet the Anti-CRISPRs: Widespread Protein Inhibitors of CRISPR-Cas Systems. CRISPR J..

[8] Rohde C., Resch G., Pirnay J.-P., Blasdel B.G., Debarbieux L., Gelman D., Górski A., Hazan R., Huys I., Kakabadze E. (2018). Expert opinion on three phage therapy related topics: Bacterial phage resistance, phage training and prophages in bacterial production strains. Viruses.

[9] Betts A., Vasse M., Kaltz O., Hochberg M.E. (2013). Back to the future: Evolving bacteriophages to increase their effectiveness against the pathogen Pseudomonas aeruginosa PAO1. Evol. Appl..

[10] Friman V.P., Soanes-Brown D., Sierocinski P., Molin S., Johansen H.K., Merabishvili M., Pirnay J.P., De Vos D., Buckling A. (2016). Pre-adapting parasitic phages to a pathogen leads to increased pathogen clearance and lowered resistance evolution with Pseudomonas aeruginosa cystic fibrosis bacterial isolates. J. Evol. Biol..

[11] Declercq A.M., Haesebrouck F., Van den Broeck W., Bossier P., Decostere A. (2013). Columnaris disease in fish: A review with emphasis on bacterium-host interactions. Vet. Res..

[12] Laanto E., Bamford J.K.H., Laakso J., Sundberg L.-R. (2012). Phage-driven loss of virulence in a fish pathogenic bacterium. PLoS ONE..

[13] Penttinen R., Hoikkala V., Sundberg L.-R. (2018). Gliding Motility and Expression of Motility-Related Genes in Spreading and Non-Spreading Colonies of Flavobacterium columnare. Front. Microbiol..

[14] León M., Bastías R. (2015). Virulence reduction in bacteriophage resistant bacteria. Front Microbiol..

[15] Laanto E., Hoikkala V., Ravantti J., Sundberg L.-R. (2017). Long-term genomic coevolution of host-parasite interaction in the natural environment. Nat. Commun..

[16] Hoikkala V., Ravantti J.J., Diez-Villasenor C., Tiirola M., Conrad R., McBride M.J., Sundberg L.R. (2020). Cooperation between CRISPR-Cas types enables adaptation in an RNA-targeting system. BioRxiv.

[17] Laanto E., Sundberg L.-R., Bamford J.K.H. (2011). Phage specificity of the freshwater fish pathogen Flavobacterium columnare. Appl. Environ. Microbiol..

[18] Sundberg L.-R., Kunttu H.M.T., Valtonen E.T. (2014). Starvation can diversify the population structure and virulence strategies of an environmentally transmitting fish pathogen. BMC Microbiol..

[19] Weitz J.S., Hartman H., Levin S.A. (2005). Coevolutionary arms races between bacteria and bacteriophage. Proc. Natl. Acad. Sci. USA.

[20] Stern A., Sorek R. (2011). The phage-host arms race: Shaping the evolution of microbes. Bioessays.

[21] Górski A., Międzybrodzki R., Węgrzyn G., Jończyk-Matysiak E., Borysowski J. (2020). Weber-Dąbrowska, B. Phage therapy: Current status and perspectives. Med. Res. Rev..

[22] Laanto E., Bamford J.K.H., Ravantti J.J., Sundberg L.-R. (2015). The use of phage FCL-2 as an alternative to chemotherapy against columnaris disease in aquaculture. Front Microbiol..

[23] Almeida G.M.F., Laanto E., Ashrafi R., Sundberg L.-R. (2019). Bacteriophage Adherence to Mucus Mediates Preventive Protection against Pathogenic Bacteria. MBio.

[24] Lopez-Pascua L.d.C., Buckling A. (2008). Increasing productivity accelerates host-parasite coevolution. J. Evol. Biol..

[25] Harrison E., Brockhurst M.A. (2017). Ecological and evolutionary benefits of temperate phage: What does or doesn’t kill you makes you stronger. Bioessays.

[26] Bohannan B.J.M., Lenski R.E. (2000). The Relative Importance of Competition and Predation Varies with Productivity in a Model Community. Am. Nat..

[27] Harrison E., Laine A.-L., Hietala M., Brockhurst M.A. (2013). Rapidly fluctuating environments constrain coevolutionary arms races by impeding selective sweeps. Proc. Biol. Sci..

[28] Van Houte S., Ekroth A.K.E., Broniewski J.M., Chabas H., Ashby B., Bondy-Denomy J., Gandon S., Boots M., Paterson S., Buckling A. (2016). The diversity-generating benefits of a prokaryotic adaptive immune system. Nature.

[29] Meeske A.J., Nakandakari-Higa S., Marraffini L.A. (2019). Cas13-induced cellular dormancy prevents the rise of CRISPR-resistant bacteriophage. Nature.

[30] Nelson S.S., Bollampalli S., McBride M.J. (2008). SprB is a cell surface component of the Flavobacterium johnsoniae gliding motility machinery. J. Bacteriol..

[31] Shrivastava A., Rhodes R.G., Pochiraju S., Nakane D., McBride M.J. (2012). Flavobacterium johnsoniae RemA is a mobile cell surface lectin involved in gliding. J. Bacteriol..

[32] Kunttu H.M.T., Sundberg L.-R., Pulkkinen K., Valtonen E.T. (2012). Environment may be the source of Flavobacterium columnare outbreaks at fish farms. Environ. Microbiol. Rep..

[33] Zhang J., Laakso J., Mappes J., Laanto E., Ketola T., Bamford J.K.H., Kunttu H., Sundberg L.R. (2014). Association of colony morphotypes with virulence, growth and resistance against protozoan predation in the fish pathogen Flavobacterium columnare. FEMS Microbiol. Ecol..

[34] Hyman P., van Raaij M. (2018). Bacteriophage T4 long tail fiber domains. Biophys. Rev..

[35] Islam M.Z., Fokine A., Mahalingam M., Zhang Z., Garcia-Doval C., van Raaij M.J., Rossmann M.G., Rao V.B. (2019). Molecular anatomy of the receptor binding module of a bacteriophage long tail fiber. PLoS Pathog..

[36] Tétart F., Repoila F., Monod C., Krisch H.M. (1996). Bacteriophage T4 host range is expanded by duplications of a small domain of the tail fiber adhesin. J. Mol. Biol..

[37] Sergueev K.V., Filippov A.A., Farlow J., Su W., Kvachadze L., Balarjishvili N., Kutateladze M., Nikolich M.P. (2019). Correlation of Host Range Expansion of Therapeutic Bacteriophage Sb-1 with Allele State at a Hypervariable Repeat Locus. Appl. Environ. Microbiol..

[38] Gallet R., Lenormand T., Wang I.-N. (2012). Phenotypic stochasticity protects lytic bacteriophage populations from extinction during the bacterial stationary phase. Evolution.

[39] Holtzman T., Globus R., Molshanski-Mor S., Ben-Shem A., Yosef I., Qimron U. (2020). A continuous evolution system for contracting the host range of bacteriophage T7. Sci. Rep..

[40] Dunne M., Rupf B., Tala M., Qabrati X., Ernst P., Shen Y., Sumrall E., Heeb L., Plückthun A., Loessner M.J. (2019). Reprogramming Bacteriophage Host Range through Structure-Guided Design of Chimeric Receptor Binding Proteins. Cell Rep..

[41] Kunttu H.M.T., Suomalainen L.-R., Jokinen E.I., Valtonen E.T. (2009). Flavobacterium columnare colony types: Connection to adhesion and virulence?. Microb. Pathog..

[42] Song Y.L., Fryer J.L., Rohovec J.S. (1988). Comparison of six media for the cultivation of Flexibacter columnaris. Fish Pathol..

[43] Kropinski A.M. (2009). Measurement of the rate of attachment of bacteriophage to cells. Methods Mol. Biol..

[44] Edgar R.C. (2004). MUSCLE: Multiple sequence alignment with high accuracy and high throughput. Nucleic Acids Res..

